# Medical News and Miscellany

**Published:** 1896-04

**Authors:** 


### Medical News and Miscellany.

**Dr. B. H. Rand** has removed from Allenfarm to Pittsburg, Texas.

**Our friend** Dr. "Jack" Sampson, has removed from Galveston to Houfton.

**Prof. Lemond,** of Gross Medical College, formerly a Texas practitioner at Vernon, Texas, will attend the Fort Worth meeting on the 28th and read a paper.

**Our** esteemed contemporary Bell, of the *Southwestern Medi-*

*cal Reporter*, is taking a special course at New Orleans Polyclinic. He will be on hand at Fort Worth, however.

---

**Dr. D. F. Kirkpatrick,** of Waketon, Texas, has been spending several months in New Orleans attending the Medical Department of Tulane University and the New Orleans Polyclinic.

---

**Dr. Wm. Gammon,** late of the Sealy Hospital, Galveston, has gone to New York, whence he will sail on the 12th inst. for Paris, to perfect his studies in bacteriology, microscopy and pathology.

---

**Erratum.**—The JOURNAL was mistaken in saying that Prof. Geo. Dock, of the Michigan University Medical College, is an alumnus of Jefferson Medical College. He is from the University of Pennsylvania.

---

**Subscribers** will please note that bills have been sent out with this issue to all who are in arrears, and all whose subscription has expired. We solicit renewal of one and payment of the other, and will be gratified if our friends will go to Fort Worth prepared to attend to the matter when they see the Red Back there and its representative.

---

**Beware!**—A respectable doctor in one of the interior Texas towns prescribed whisky for a patient (it is a "dry" town). The ladies (?) of the place, to the number of twenty-five, demanded he should leave the county. Refusing, he was horsewhipped unmercifully. It is gratifying to learn the "ladies" were arrested and placed in the court, and will be tried for assault.

---

**Dr. W. D. Yett,** of Marble Falls, is a candidate for State Senator from this district—Travis, Williamson and Burnet counties. Good. Hope he may be elected, for we surely want intelligent medical men in the Senate. We are going to ask for a bill to regulate the practice, and a doctor can appreciate the necessity for it, and doubtless influence others to vote for it.

---

**We will** just have to kill him; there is no alternative. Dr. Merriman now writes the JOURNAL, "to ask Dan'els," he says, "if the late William E. (Bill) Nye, was any relation to *Sphincter A.* ni? and if so, what?"

The JOURMAL is not prepared to say, but we suppose there must have been some relation, of a fundamental character, between the two.

**Dr. W. N. Rogers,** of Belton, the JOURNAL regrets to learn, is in failing health, and has gone north to consult specialists. He will visit St. Louis and New York. Should his health permit, later, he will take a special course of instruction in the Polyclinic. In the meantime, we learn, that Dr. J. D. Law, of Salado, has removed to Belton and will take charge of Dr. Rogers' practice, forming a copartnership upon Dr. Rogers' return.

**Dr. C. M. Rosser,** Superintendent North Texas Insane Asylum, by invitation, recently, delivered a course of six lectures on insanity to the class of Gross Medical College, Denver, and was presented by the students with a handsome gold watch, accompanied by a scroll engraved, as a memento of the occasion. Dr. Rosser delivered the alumni address for the Medical Department, University of Louisville, at its recent commencement. He roasted the homeopaths, they say.

**Mal-apropos.**—In our March number there was mention of a "new cure" in Japan, and when we came across another "new cure," this time in South America. we made a paragraph of it also, heading it "apropos;" it being "apropos" of the subject of cures, etc. Greatly to our vexation the intelligent compos. (*non*-compos) separated the paragraphs, and thus got the S. A. "new cure" apropos of Dr. Dock and the University of Michigan! Confound the printer, for so confounding things and "us."

**Wanted, a Diagnosis.**—*Of course*, all doctors have read that most charming of all books—the more charming by contrast with the dirty literature of the day—"Under the Bonny Brier Bush," by Ian MacLaren. (By the bye, don't you spell brier-bush—bri*a*r?) Well, what was the matter with Annie Mitchell, on whom the great London surgeon, Sir George (who weakened on crossing the Totchty) operated? Don't all speak at once, but we would like to get a consensus of medical opinion as to the diagnosis.

**Attention** is called to the announcement in this issue, of a special course in bacteriology and microscopy at Galveston by Prof. A. J. Smith, M. D., during May and June. It offers a splendid opportunity to Texas physicians to acquire a thorough knowledge of these branches at home, and at a very little expense. The proceeds, too, are for the better equipment of the chair of Microscopy and Pathology of the medical department of our State University. Correspond with the doctor, and take out your ticket in time, as the class will be limited to a small number.

---

**Dr. W. A. Harper,** who for some time was teacher and superintendent of the male department of the Texas Institute for the Blind at Austin, after graduating in medicine at Sewanee Medical College last summer, took a post-graduate course last winter at the University of Tennessee. He is now at Johns Hopkins University, taking special instruction, and will spend some weeks in the hospital and colleges in New York; after which, we learn, he will return to Austin to locate for practice. Dr. Harper is a young physician of superior attainments, for whom we predict a fine career.

---

**We are** requested by the Fort Worth Pharmacy Company, of Fort Worth, Texas, to say, that they will be pleased, upon application, to mail their Surgical Instrument Catalogue and Price List to any member of the profession.

Their catalogue quotes over six thousand articles and gives about the same number of cuts or figures of the articles.

They will also be pleased, at request, to mail special catalogues of Electric Batteries, Microscopes, Physician's Chairs and Tables and compressed air machines, which are now so successfully used by specialists in the treatment of throat and catarrhal diseases.

---

**"Make Way for the Doctor!"**—The city council of Chicago "cried,"' and forthwith made way for the doctor, but didn't "die." The council passed an ordinance stipulating that doctors answering urgent calls, and ambulances going for or returning with sick or wounded shall have the right-of-way on the streets, of all processions, etc., which must turn out at sight of the insignia of authority. This is a red badge (significant of blood, perhaps,) worn on the lappel of the coat of the doctor or driver. It is said the city clerk did a land office business the first day by selling the red badges at 50 cents each. Great is Chicago! and greater her doctors!

---

T. S. MED. ASSO., OFFICE OF PRESIDENT,
COLORADO, TEXAS, April 1st, 1896.

DEAR DOCTOR:—I beg leave to call your attentlon to perhaps the most important question which will come before the State

Medical Association at our annual session in Fort Worth on the 28th instant, namely, recommendations on "the best means of increasing the membership, and thereby the usefulness of the State Medical Association."

I hope you will give the question *serious thought* before we meet, and give the Association the benefit of your conclusions.

Fraternally yours,

P. C. COLEMAN,
President T. S. M. A.

---

**National Sanitarium for Consumptives.**—A bill has been introduced in the lower house of congress to set aside Oklahoma Territory for a national sanitarium for consumptives. The dispatches only announced the fact, without stating what had become of the bill. This is a most sensible move, and should be pushed. It is to be hoped it will pass. It is a reproach to our civilization that a preventable disease should every year carry off so large a share of humanity, and be responsible for one-seventh of all the deaths. Such matters come properly within the scope and provision of state medicine, and should congress ever give us a health department, we may look for a broader intelligence in legislation than has characterized certain acts.

The *Texas Medical and Surgical Record's* attention is called to the above, as that journal hinted at the absurdity of such a thing awhile back. The unexpected sometimes happens.

---

**Asexualization** as Prentative of Disease and Crime is the title of a very excellent paper recently published by Dr. E. Stuver, of Rawlins, Wyoming, president of a large institution of learning at that place. It is one of the very best papers on the subject that has yet been written, and deals very exhaustively with the subject, especially pointing out the folly of attempting to remove the evil without taking steps to prevent the hereditary transmission. Crime, in the light of science, is becoming to be regarded as disease; disease of the social body, and the dictates of reason, humanity and common sense demand that it should be dealt with intelligently, and not indiscriminately punished. "Punishment," says an eminent medical authority, "should have no place in a civilized code, and is permissible only as a feature in necessary prison discipline, a factor in the cure or reformation of criminals.

---

**The Doctor's Lien on the Baby.**—An esteemed correspondent sends us the following: "The item in your issue of February

22d, in which the colored midwife of Gallatin, Tenn., wished to levy on the baby in order to secure the payment of her hard-earned fee, brings to mind an incident which occurred in my own practice. I attended the wife of a young woman in her first confinement, delivering her of a fine large boy. Several weeks after the father called at my office and asked for his bill, which being made out, receipted, and handed to him, he paid the money, remarking with evident satisfaction as he placed the receipt in his pocket: 'That boy belongs to me now.' It would be well for the profession if parents generally believed that the doctor had a lien on the child until the fee for the confinement is paid."—*Medical Record.*

[The rule should be C. O. D.—Ed.]

---

**The New York Medical Rip-Van-Record.**—Some time ago ye *Record* twitted "certain journals" for publishing personal items of doctors (such as marriage-death-removal, appointment, etc.) as "medical news," and said such items are not "news at all"; said the *Record*, now, always gives "news" as *is* news,—(unlike ye "certain journals";—ergo, ye *Record* stamps itself as an uncertain journal; and such it is, sometimes). For, as to "news," now: in ye *Record* of March 14, 1896 (ult.), we find the following "news":

"*Asexualization of Criminals as a Prevention of Crime.*—Dr. F. L. Sim, of Memphis, Tenn., argues ably in favor of sterilization of certain criminals in order to curtail the crime of rape.

"Executions partake of revenge and are more than the demands of society require. It would be wiser to substitute a more humane and scientific punishment, and one that would at the same time convey a sufficient object lesson."—*N. Y. Med. Record.*

The crispness and relish of the *Record's* "news" will be the more apparent when it is recalled that Dr. Sim's paper was published in August, 1894!—about a *year after* a paper, advocating the same measures and views by the editor of this Journal, had been read before the International Medico-Legal Congress, and extensively published; meantime, Dr. Sim has been *dead* over a year!

The "uncertainty" of the New York Medical *Rip-Van-Record* is forcibly exhibited in an editorial on page 379, in the March No. (ult.), where, in speaking of the address of Garofolo in

Rome, recently, the editor says: "To the religious instruction given in Great Britain and the United States he attributes the fact that these countries have, in forty years, diminished by one-half, their annual proportion of delinquents and mendicants."

If there is one fact well established in criminalogy, it is that crime is greatly on the increase both in England and the United States. In the latter, according to the United States census—while, from 1850 to 1890, the population has increased 170 per cent, the criminals have increased 445 per cent; and in the decade, 1880 to 1890, the criminal element was more than doubled!

*Wake up!* old man.

---

**For the meeting** of the State Medical Association, at Fort Worth, on April 28th to May 1st, the *Gulf*, *Colorado & Santa Fe* will make reduced rates on the certificate plan from all points on that line.

Delegates and others attending this meeting, will purchase single trip tickets, paying local one way rate, obtaining a certificate from the selling agent, which is in the forn of a receipt for the ticket purchased. This receipt, when signed by Dr. H. A. West, Secretary, and countersigned by the joint agent at Fort Worth who will be appointed for this occasion, will be authority for the agent at Fort Worth to sell the return ticket to destination at one-third of the one-way rate, provided that the north bound trip is made not earlier than April 27th, and that the receipt is presented for return ticket not later than May 2d, or twenty-four hours after adjournment of the meeting. Further, provided there are fifty or more people in attendance upon this meeting.

The Santa Fe operate double daily trains on its main line to and from Fort Worth. Free reclining chair cars and Pullman palace sleepers.

Apply to any agent of the Santa Fe line for further information, or address, W. S. KEENAN,
G. P. A., Galveston.

---

**Weir's Index to the Medical Press.**—Frank Weir & Co., of New York, will issue, about 15th April, the first number of this work; they announce that:

"The initial and each successive issue will treat the entire medical literature of the month immediately preceding as one vast volume, to which it will aim to be the *Index* or *Contents Table*. For this purpose an editorial staff—the personnel of which has been carefully chosen, in order to assure prompt and accurate work—will review monthly the entire medical press of the United States and Canada, including, in addition to the Published Transactions of the various National and State Medical Societies, the current number of every important Medical periodical published in the two countries. The result of its labors will be presented in the form of a Monthly Magazine of from 112 to 128 pages, to be known as *Weir's Index to the Medical Press*.

"For greater facility of reference, the Science of Medicine will be divided into its various departments, and under appropriate sub-heads will be briefly indexed, in alphabetical order, every Leading Article, Reported Case, etc., etc., found in the current press, together with the name of its author or contributor, the approximate space it occupies, and the title, serial number and cost of the periodical in which it is to be found.

"Each monthly part will thus be a Reference Work of constant value to the author, professor, and specialist—as well as to the general practitioner—enabling him to follow closely the general trend of Medical Science, and avail at once of any matter of special interest to him. To facillitate this latter desideratum still further we will constitute ourselves a Depot or Supply House for all Indexed publications, so that subscribers, desiring to purchase single copies of more than one publication at the one time may, by a single order addressed to us, avoid the necessity of communicating with each publisher direct. The price will be $3.00 per annum."

The "Red Back" has arranged with the publishers to forward the initial number of the *Index*, without charge, to all our subscribers, provided they send in a request for same prior to April 15th, mentioning the JOURNAL.

---

**Epitaph of a Noble Life.**—[Written in memory of a brave yellow fever nurse.] Holly Spring, Miss., March 21, 1896.—On the north wall of the jury room of the court house is written with a lead pencil an epitaph of one of the noblest lives ever sacrificed for the good of humanity, and which serves to recall one of the darkest pages in the history of Holly Springs. During the terrible yellow fever epidemic of 1878, when the saffron king claimed his victims by hundreds, the court house was used as a hospital. Into this house of death came the Roman Catholic sisters of Bethlehem Academy to minister to the sick and dying. About twelve came, and of this number seven died. Beneath the inscription in the jury room is written, "Let no one deface this," and though the walls are black with the scribbling of idle jurors, the inscription has never been touched. It was writteu by Dr. R. M. Swearingen, of Austin, Texas, who volunteered his services at the outbreak of the fever, and reads as follows:

"Within this room, September, 1873, Sister Corintha sank into the sleep eternal. Among the first to enter this realm of death, she was the last, save one, to leave. The writer of this humble notice saw her in health, gentle but strong as she moved with noiseless step and serene smile through the crowded ward. He saw her when the yellow plumed angel threw his golden shadows over the last sad scene, and eyes unused to weeping paid the tribute of tears to the brave and beautiful 'Spirit of Mercy.'"

"She needs no slab of Parian marble,
  With its white and ghastly head,
To tell wanderers in the valley
  The virtues of the dead.
Let the lily be her tombstone,
  And the dewdrops pure and bright,
The epitaphs the angels write
  In the stillness of the night."

—*Memphis Commercial-Appeal.*

---

### CIRCULAR TO PHYSICIANS.

### Eye and Ear Charity Hospital.

Through the co-operation of some of the good women of Texas, there has been founded in the city of Austin an Eye and Ear Charity Hospital for patients who are unable to pay for board and treatment. Arrangements have also been made to receive and treat patients who are able to pay. Only trained nurses are employed, and the surgeon in charge is daily assisted by Dr. Jos. S. Wooten.

I take this method of announcing to the profession that the hospital was opened September 1st, 1895, and has been in operation since that date. Your co-operation is respectfully solicited, and a cordial invitation is extended to you, when in the city, to visit the hospital and see for yourself that it is properly conducted.

For further information address

H. L. HILGARTNER, M. D.,
Surgeon in Charge.

